# Wildebeest-associated malignant catarrhal fever: perspectives for integrated control of a lymphoproliferative disease of cattle in sub-Saharan Africa

**DOI:** 10.1007/s00705-015-2617-6

**Published:** 2015-10-08

**Authors:** Lillian Wambua, Peninah Nduku Wambua, Allan Maurice Ramogo, Domnic Mijele, Moses Yongo Otiende

**Affiliations:** School of Biological Sciences, University of Nairobi, P.O Box 30197, 00100 Nairobi, Kenya; International Center for Insect Physiology and Ecology, P.O Box 30772, 00100 Nairobi, Kenya; Kenya Wildlife Service, P.O Box 40241, 00100, Nairobi Kenya

## Abstract

Wildebeest-associated malignant catarrhal fever (WA-MCF), an acute lymphoproliferative disease of cattle caused by alcelaphine herpesvirus 1 (AlHV-1), remains a significant constraint to cattle production in nomadic pastoralist systems in eastern and southern Africa. The transmission of WA-MCF is dependent on the presence of the wildlife reservoir, i.e. wildebeest, belonging to the species *Connochaetes taurinus* and *Connochaetes gnou*; hence, the distribution of WA-MCF is largely restricted to Kenya, Tanzania and the Republic of South Africa, where wildebeest are present. WA-MCF is analogous to sheep-associated MCF (SA-MCF) in many aspects, with the latter having sheep as its reservoir host and a more global distribution, mainly in developed countries with intensive livestock production systems. However, unlike SA-MCF, the geographic seclusion of WA-MCF may have contributed to an apparent neglect in research efforts aimed at increased biological understanding and control of the disease. This review aims to highlight the importance of WA-MCF and the need for intensified research towards measures for its integrated control. We discuss current knowledge on transmission and geographical distribution in eastern and southern Africa and the burden of WA-MCF in affected vulnerable pastoral communities in Africa. Recent findings towards vaccine development and pertinent knowledge gaps for future research efforts on WA-MCF are also considered. Finally, integrated control of WA-MCF based on a logical three-pronged framework is proposed, contextualizing vaccine development, next-generation diagnostics, and diversity studies targeted to the viral pathogen and cattle hosts.

## Introduction

Malignant catarrhal fever (MCF), also referred to as African malignant catarrhal fever, bovine malignant catarrhal fever, or *Snotsieke* is a collective term for the clinicopathological signs manifested by cattle and other susceptible ungulates when infected with viruses of the genus *Macavirus* of the subfamily *Gammaherpesvirinae* [1]. Two viruses are important with respect to MCF in cattle; alcelaphine herpesvirus 1 (AlHV-1) and ovine herpesvirus 2 (OvHV-2). The natural hosts for AlHV-1 are blue and black wildebeest (*Connochaetes taurinus* and *Connochaetes gnou*, respectively). Sheep (*Ovies aries*), on the other hand, are the natural hosts for OvHV-2. Cattle are susceptible to both AlHV-1 and OvHV-2. Infection of cattle with AlHV-1 results in wildebeest-associated MCF (WA-MCF), while OvHV-2 causes sheep-associated MCF (SA-MCF). Each virus is adapted to its natural host, therefore causing inapparent infection in that species. WA-MCF in cattle mainly occurs in Kenya, Tanzania and South Africa and is restricted to specific geographical zones and private conservancies where the susceptible cattle interact with wildebeest, which are asymptomatic carriers of AlHV-1. Unless otherwise stated, this review focuses on WA-MCF in eastern and southern Africa.

## Etiology of WA-MCF

The history of WA-MCF arguably dates back as far as the co-existence of cattle, wildebeest and other natural hosts. However, the earliest and most comprehensive report of WA-MCF in sub-Saharan Africa was that by Plowright and colleagues [2], which described AlHV-1 as the etiological agent of WA-MCF and the blue wildebeest as the natural host for the virus in East Africa. Previous studies on the natural hosts indicate that almost 100 % of wildebeest are asymptomatic carriers of AlHV-1 [3]. In wildebeest, newborn calves may acquire AlHV-1 infection either prenatally through congenital transmission *in utero* from infected dams [4] or perinatally by interaction with other infected calves shedding high titers of the cell-free virus in their ocular and nasal secretions [5]. The infected calves subsequently acquire neutralizing antibodies to the virus and remain latently infected throughout their lives [6]. As with other herpesviruses, AlHV-1 may be reactivated in adult wildebeest if their immunological competence is compromised by pregnancy or by stressful conditions such as captivity or starvation, thereby rendering them infective to cattle and other susceptible hosts [7].

Transmission of AlHV-1 to cattle occurs when they come into close contact with wildebeest calves shedding cell-free viruses in their ocular and nasal secretions [8]. The respiratory tract is the natural route of infection. There is no documented evidence of horizontal transfer of AlHV-1 from infected cattle to uninfected cattle, and hence, infected cattle are terminal/dead-end hosts of AlHV-1. However, vertical transmission of the virus also occurs in cattle, as in wildebeest, where the infected cow transmits the virus transplacentally to her unborn foetus in the course of gestation [9].

Contact of cattle with fetuses and placental material of calving wildebeest has been advanced as another possible mode of transmission of AlHV-1 [10]. This mode of transmission was based on the demonstration of cell-free virus in unborn fetuses and placental tissue of calving wildebeest. Although previous studies by Rositter and co-workers [11] indicated that the virus was not present in fetal tissue and fluids, Lankester *et al.* [5] demonstrated the presence of AlHV-1 viral DNA in 50 % of placentae of calving wildebeest in Tanzania. Therefore, transmission of AlHV-1 from calving wildebeest to cattle through contact with fetal and placental material may be a possible mode of infection, and the physical presence of these tissues in rangelands should be viewed as visual indicators of newborn wildebeest calves, representing a real threat of infection to cattle.

The involvement of a vector or intermediate host in the transmission of AlHV-1 from wildebeest to cattle remains largely speculative. Barnard and co-workers [12] observed transmission of AlHV-1 in northwestern Transvaal (presently Northwest Province), South Africa. In that study, the incidence of WA-MCF in cattle was negatively correlated with the proximity to wildebeest, and the number of cases in cattle peaked in the spring season, during which wildebeest calves under the age of 3 months were absent. Since young calves (< 3 months) are implicated in active transmission of AlHV-1, their absence was suggestive of possible involvement of an intermediate host or vector in the transmission cycle of WA-MCF to cattle. Subsequent investigations, however, failed to find evidence that arthropod vectors are involved in the spread of the virus [13]. Hence, to date, there remains no substantive evidence of an intermediate vertebrate or invertebrate host in the transmission of AlHV-1 from wildebeest to cattle.

As with other herpesviruses, the exact incubation period of AlHV-1 in cattle and other experimental hosts is uncertain. Mushi et al. [14] reported a mean incubation period of 14 days in rabbits infected with AlHV-1, while Jacoby et al. [15] reported 21–90 days in rodent models of AlHV-1. In cattle, a mean incubation period of 16–29 days was achieved in an experimental infection with AlHV-1 [16], while in a more recent experimental investigation, the observed incubation period ranged between 21 and 68 days [17]. Based on these reports, the incubation period of AlHV-1 in susceptible hosts is arguably a function of the virus titer upon infection, host immunity, the route of inoculation, or other factors. However, 95 %–100 % of affected cattle die within 4–7 days of the onset of clinical signs. Recovery of cattle from natural infection of WA-MCF is not fully understood or substantially documented, although there are existing reports of recovery from experimental infection [9].

## The WA-MCF landscape in eastern and southern Africa

At present, WA-MCF remains a serious cause of human-wildlife conflict between pastoralists and wildlife custodians in the vast rangelands of sub-Saharan Africa. The disease is largely restricted to wildebeest zones: commonly, open savannah grasslands, which are home to large populations of wildebeest, other wild ungulates and livestock. Cattle and wildebeest are herbivores, exhibiting over 70 % overlap in nutritional requirements and dietary preferences [18], which favors close inter-species interactions, facilitating transmission of WA-MCF. Peak transmission of WA-MCF occurs during the highly synchronized, annual wildebeest calving season. This season is characterized by migration of large herds of wildebeest to their preferred calving zones in the savannah plains, where they encounter cattle grazing in the lush pastures of the plains. The calving sites inevitably become hotspots for WA-MCF transmission.

## Epidemiology of WA-MCF in eastern Africa

**Fig. 1:**
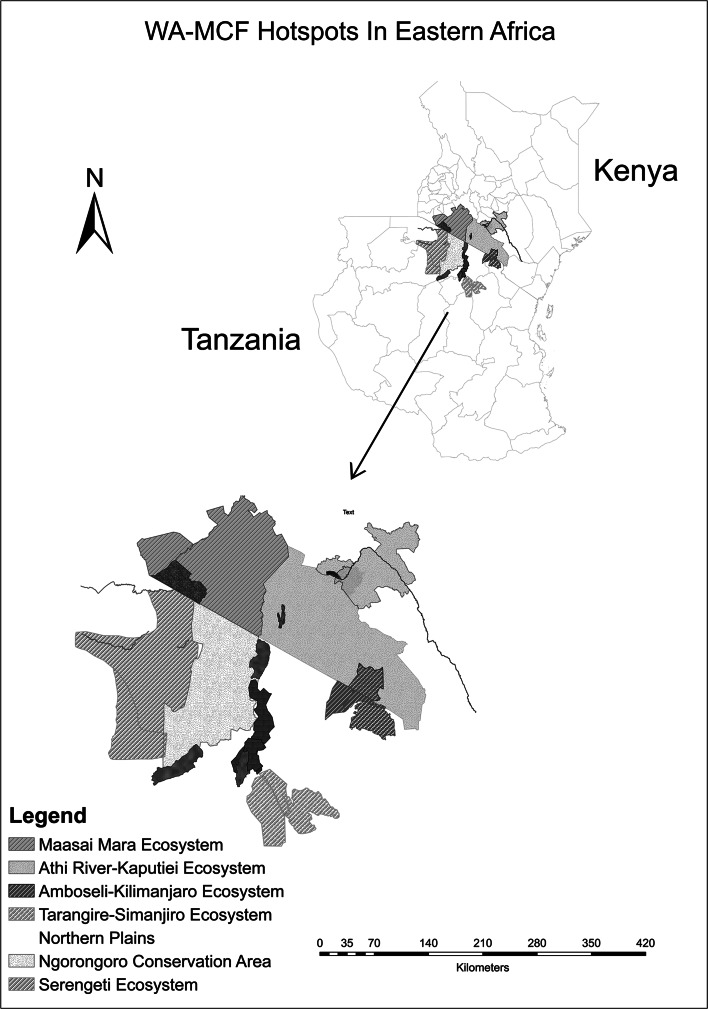
WA-MCF landscape in eastern Africa: maps indicating the transmission hotspots of WA-MCF in Kenya and Tanzania

In eastern Africa, WA-MCF is commonly referred to by pastoralists as *“ugonjwa wa nyumbu*” a Swahili term directly translated to mean “disease of the gnu or wildebeest.” It is the most important cattle disease with the highest perceived impact on cattle production and livelihoods of pastoralist communities [19]. The principal reservoirs of WA-MCF in East Africa are the blue wildebeest, *Connochaetes taurinus*. Annual outbreaks of WA-MCF occur in the wildebeest zones in Kenya and Tanzania, coinciding with the wildebeest calving season. Peak transmission is reported between February and April in Tanzania, varying slightly to March and June in Kenya, every year [20]. Figure 1 summarizes WA-MCF hotspots in eastern Africa.

In Kenya, the WA-MCF landscape can be mapped onto three principal wildebeest zones, located in the southwestern region of the country. They include i) the Maasai Mara ecosystem, comprising of the Maasai Mara National Reserve, associated private group ranches, and surrounding areas of Narok county stretching into Serengeti in Tanzania; ii) the Athi-Kaputiei ecosystem comprising of the Nairobi National Park, Kitengela Game Conservation Area, and Athi-Kaputiei plains, currently located in the larger administrative areas of Kajiado and Machakos counties; and iii) the Amboseli-Kilimanjaro ecosystem, comprising the Amboseli National Park and associated group ranches, stretching into Mt. Kilimanjaro in Tanzania [21–23].

In Tanzania, the WA-MCF landscape is found in the northern region of the country and can be mapped onto four principal zones: i) the Serengeti ecosystem contiguous with the Maasai Mara ecosystem in southern Kenya, encompassing the Serengeti National Park, Maswa Game Reserve and the Serengeti plains; ii) the Ngorongoro conservation area, extending to Loliondo, a known wildebeest calving area, and the Angata Kheri and Salei plains, the latter of which are documented as a recent extension of the wildebeest zone in Ngorongoro due to an increase in migratory populations of wildebeest [20]; iii) the Manyara-Lake Natron ecosystem, mainly the northern plains stretching north of Tarangire National Park (TNP), through Manyara ranch to the shores of Lake Natron; and finally, iv) the Tarangire-Simanjiro ecosystem in the Maasai Steppe, including Tarangire National Park and stretching eastward to the Simanjiro plains, a dispersal and calving area for wildebeest and other wildlife species.

## Epidemiology of WA-MCF in southern Africa

In southern Africa, WA-MCF occurs in the Republic of South Africa, Namibia, Zimbabwe, Zambia and Botswana, with significant impact mainly on commercial cattle production systems and, to a lesser extent, smallholder systems. Although WA-MCF is widely documented in South Africa, it is equally of significant importance in Namibia, Zambia, Zimbabwe and Botswana, where there are substantial populations of wildebeest [24]. A recent outbreak in Zimbabwe, where WA-MCF accounted for 71 % and 21 % losses of cattle reared under commercial and smallholder production systems respectively, demonstrated the importance of the disease in the southern Africa region [25].

The epidemiology of WA-MCF in southern Africa tends to differ from that in eastern Africa, where, two annual outbreaks are observed [25, 26]. The first outbreak occurs early in the year as calving season of the wildebeest starts in December. WA-MCF cases begin to be reported in January and peak in March and April, as in Tanzania and Kenya. However, a second annual outbreak, where the majority of the cases are observed, occurs in the months of September to November when wildebeest calves are 8-10 months old. This outbreak is hypothesized to be attributed to stress in cattle caused by unusually cold rainy weather during the winter months, coupled with poor grazing conditions [27].

In South Africa, cases of WA-MCF have been reported with increasing frequency as a result of growth in game ranching, wildlife and the tourism industry [27]. The majority of cases occur in Limpopo and Northwest provinces, where the number of game ranches have increased. Substantial losses have been reported in the Northwest Province, with incidences as high as 34 % in farms adjacent to game reserves. Black wildebeest (*Connochaetes gnou*) play an important role in the epidemiology of WA-MCF in South Africa, unlike in eastern Africa, where blue wildebeest (*Connochaetes taurinus*) are regarded as the principal natural hosts [3]. A comparison of outbreaks associated with black wildebeest in South Africa between the periods 1981–1983 and 1988–1990 revealed that the number of cases of WA-MCF where only black wildebeest were involved had increased 7-fold [28] due to growth in the number of farms on which black wildebeest were kept.

Annual incidence of WA-MCF is highly variable. In Kenya, 1 %-21 % annual incidence was estimated by Ngotho and co-workers [29], whereas Bedelian et al. [19] estimated the incidence to be 3 %-12 % in the year 2003-2004. In Tanzania’s Ngorongoro area, the mean incidence for the year 2000 was estimated at 5.6–6.2 % [20]. Higher annual incidence of WA-MCF has been reported in South Africa, rising up to 34 % due to an increase in game farming and ecotourism [26, 30]. An upward trend in the number of outbreaks has been noted in recent years, with up to 50 outbreaks of WA-MCF being reported per annum in sub-Saharan Africa [31, 32].

The true incidence of WA-MCF is higher than current estimations due to widespread underreporting and misdiagnoses. Nevertheless, several factors may account for the high variation in reported annual incidence of WA-MCF in sub-Saharan Africa. Annual rainfall levels, proximity of cattle populations to wildebeest calving areas, density of wildebeest in the area and host genetics may account for the observed variation in annual incidence of WA-MCF. The highest incidence of the disease occurs in the cattle populations with the nearest proximity to the wildebeest calving areas and is positively correlated with density of wildebeest interacting with cattle populations [19]. WA-MCF incidence is also associated with decreased annual rainfall, decrease in browser vegetation and drought, as these adverse conditions discourage the movement of wildebeest to their usual calving zones, thereby reducing transmission of WA-MCF [33]. The increase in human populations, coupled with agricultural intensification, has resulted in invasion and human settlement in rangelands originally reserved for wildlife. Encroachment on wildebeest zones and migratory routes has been noted [34]. These trends may translate to an expansion, rather than a contraction, of the WA-MCF landscape in sub-Saharan Africa, as the present realities of climate change, industrialization and increased pressure on existing land resources portend an inevitable increase in human-wildlife-livestock conflict for the limited pastures, providing ideal conditions for WA-MCF transmission.

## Contextualizing the burden of WA-MCF in affected countries

The case fatality rate of WA-MCF in affected cattle is 95-100 %. This annual catastrophic loss of cattle through WA-MCF compromises the livelihoods of affected nomadic pastoralist communities in eastern and southern Africa. Significant losses of cattle by commercial farmers are a major concern in southern Africa, where cattle are kept under intensive commercial systems for beef production [25, 30]. Furthermore, the localized mass loss of livestock resources due to WA-MCF may pose significant population bottlenecks and subsequent loss of valuable diversity. The loss of livestock genetic resources is of particular concern in Africa, where there is marked geographical segregation of locally adapted cattle breeds, whose genetic conservation is critical [35].

To date, there is no effective treatment or vaccine for WA-MCF, and field surveillance in the hotspot areas remains poor. Avoidance of interaction between wildebeest carriers and cattle remains the only control strategy for WA-MCF in the affected countries [20]. Although WA-MCF is not classified by the World Organization for Animal Health (OIE) as a transboundary or notifiable disease (2015), the disease is of significant national importance in Kenya, Tanzania, South Africa, Namibia, Zimbabwe, Botswana and Zambia, especially in areas where cattle share grazing sites with wildebeest. The disease has direct impact not only on commercial farmers but also smallholder farmers whose economic livelihoods and sociocultural lives are affected by the death of their cattle, resulting in loss of income from sale of milk and beef [19, 25].

Limited options are available for mitigation of the damage caused by WA-MCF. Cattle owners adopt either disposal or avoidance as a strategy. If infection has occurred, the pastoralists are forced to dispose off the symptomatic cattle quickly for slaughter at a throwaway price, usually 30 % or less of that of healthy cattle [19, 20]. As a preventive strategy, pastoralists avoid grazing their animals in wildebeest zones during the calving seasons and instead find pastures in thickets and highlands. Avoidance is a costly strategy, because, although the cattle are protected from WA-MCF, they are exposed to tsetse flies and ticks, further predisposing the cattle to deadly vector-borne diseases, mainly trypanosomosis and east coast fever, which may cause death, loss of productivity, and demand for costly veterinary care [19]. In eastern Africa, mobility of cattle from wildebeest zones over long distances, sometimes over 60 km, affects their body condition and productivity, reducing milk yield by up to 64 %, further increasing the economic vulnerability of pastoralist communities [36].

WA-MCF also threatens the conservation of wildebeest zones in the affected countries. It remains a hindrance in fostering community-led efforts for sustainable conservation of the dwindling wildebeest populations, since their mere existence is equated by livestock farmers to death of cattle [34]. Yet, in countries such as Kenya and Tanzania, wildebeest are keystone wildlife species, and their spectacular annual migration across the Maasai Mara-Serengeti corridor, often termed the “8th wonder of the world,” is a global tourism attraction generating millions of foreign income to the countries. WA-MCF threatens establishment of community conservancies in wildebeest dispersal areas and migratory corridors and curtails growth of an ecotourism industry that has the potential to support vulnerable communities through payment of ecosystems services and other incentives [37].

## Integrated control of WA-MCF: current knowledge and perspectives for future research

**Fig. 2:**
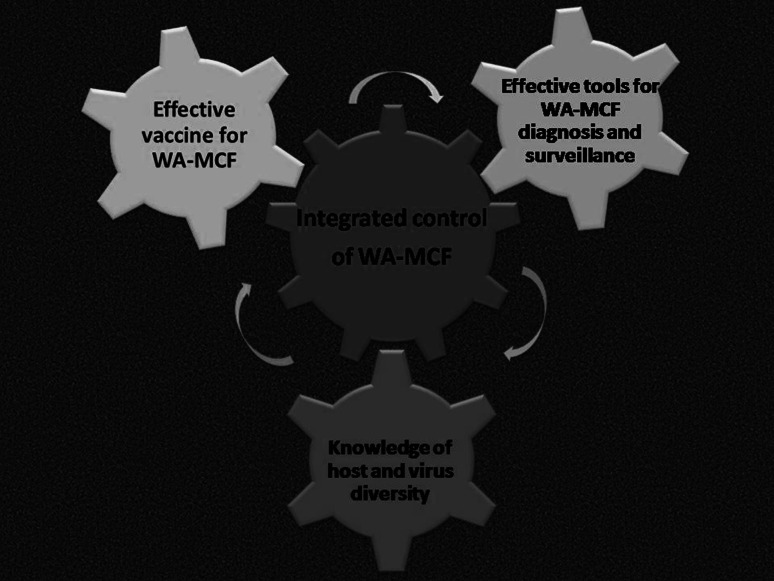
Proposed 3-pronged framework for integrated control of WA-MCF in eastern and southern Africa. Contextualizing vaccine development, next-generation diagnostic technologies and virus and host diversity in a multi-dimensional strategy for surveillance and control of WA-MCF

The control of WA-MCF to date remains largely incidental, and numerous knowledge gaps on the disease exist, which demand concerted research efforts to be addressed and a strategy for integrated control. In view of the current knowledge and with reference to steps taken to control other infectious diseases, we have proposed a three-pronged framework for the integrated control WA-MCF, encompassing vaccine development, efficient diagnostics and genetic studies of WA-MCF, as summarized in Figure 2 and discussed in detail in the sections below.

### Next-generation diagnostics for WA-MCF

MCF viruses are not readily or rapidly diagnosed by conventional virus isolation procedures. Confirmative diagnosis of WA-MCF relies mainly on postmortem histopathological analysis of samples from dead cattle, as this is the definitive test recommended by the OIE [38]. Serological tests for detection of MCF antibodies have been developed, including a competitive inhibition ELISA (CI-ELISA) [39] and a direct ELISA assay [40]. Nevertheless, their utility in routine diagnosis of WA-MCF cases is limited by the rapid and high case fatality rates associated with AlHV-1 infection, because most cattle die before a detectable antibody response has been raised [20]. These serological tests also exhibit considerable cross-reactivity with other macaviruses and must be used with caution, as they may not differentiate between AlHV-1 and OvHV-2 infections. Several PCR-based assays for detection of AlHV-1 DNA have also been developed over the years, as reviewed extensively by Li et al. [41]. DNA-based assays can distinguish between AlHV-1 and other macaviruses and have higher sensitivity than the ELISA assays. However, the current repertoire of diagnostic approaches for WA-MCF require elaborate protocols to be performed by trained personnel using specialized equipment, such as PCR machines, ELISA readers and microscopy apparatus. Samples from suspected cases must therefore be shipped from the field to the veterinary laboratories where the required facilities can be availed for molecular, serological and microscopy tests. This process is lengthy and costly and undoubtedly delays diagnosis and intervention during WA-MCF outbreaks.

It is worth noting that the current repertoire of diagnostic approaches do not support active field surveillance of WA-MCF in hotspot areas within the region. A robust surveillance system is highly dependent on development of diagnostic solutions for sensitive and speedy detection of the causative pathogen under field conditions, given that up to 100 % of symptomatic animals die within two weeks [20]. The lack of simple and low-cost diagnostic tests for AlHV-1 has arguably contributed to underreporting of WA-MCF cases in rural areas and may also be associated with the high variability in the annual incidence rates recorded for WA-MCF in sub-Saharan Africa. There is an urgent need to bridge this gap by developing low-cost penside diagnostic assays for rapid detection of WA-MCF. These tests should, however, allow differential diagnosis of AlHV-1 from other macaviruses and infectious diseases that exhibit clinical signs similar to those of WA-MCF, such as bluetongue, bovine mucosal disease and infectious bovine rhinotracheitis [42]. This next generation of diagnostic tests should exhibit high sensitivity, specificity, speed and ease of use. The development, deployment and adoption of such assays would significantly strengthen national surveillance systems for WA-MCF in affected countries in sub-Saharan Africa.

### Vaccine development for WA-MCF

Effective control of WA-MCF relies greatly on development of an effective vaccine to block the transmission of AlHV-1 from wildebeest to cattle. Such a vaccine would be highly desirable, as it would negate the need to separate cattle and other susceptible ungulates from wildebeest reservoirs. A successful vaccine for WA-MCF would therefore assure pastoralists of quality nutrition for their cattle all year round and alleviate the risk of acquiring vector-borne diseases, thereby optimizing milk and meat productivity and guaranteeing the economic livelihoods of both smallholder and commercial cattle farmers [36]. In the wider context, the availability of a WA-MCF vaccine would contribute to the conservation of the rich genetic resource of locally adapted indigenous cattle breeds present in eastern and southern Africa through protection of the animals against WA-MCF, vector-borne diseases and starvation. Hence, intensified research and efforts towards an effective WA-MCF vaccine is justified.

Concerted efforts to develop an effective vaccine for WA-MCF have been made over the years. Table 1 provides a summary of some of the key research efforts that have been made in vaccine development for WA-MCF over the last six decades. However, these efforts have so far not translated to a commercial vaccine for use by pastoralists. Initial attempts to develop a WA-MCF vaccine did not provide substantial protection against natural infection with virulent AlHV-1 [43–45]. In one study, the potential of inactivated virus as a vaccine was tested, but this failed to protect cattle from challenge with natural infection [46]. In another trial, vaccination of local animals with homologous virus isolated from cattle in America, failed to provide adequate protection against challenge despite repeated inoculations [47].

**Table 1:** Vaccine development timeline: summary of some of the key research efforts in vaccine development for WA-MCF over the last six decades, indicating the vaccine formulation, experimental hosts and outcomes

Vaccine formulation	Host tested	Year [Ref]	Outcome
Inactivated AlHV-1 virus	Rabbit and Cattle	1954 [43]	Induced neutralizing antibodies in the serum
Inactivated cell cultures of AlHV-1 (WC11 strain) in Freund’s incomplete adjuvant	Cattle	1975 [46]	High levels of neutralizing antibodies induced. No protective immunity to parenteral or natural challenge with virulent virus
Inactivated cell-free AlHV-1 virus in Freund’s complete adjuvant	Rabbit	1980 [44]	High levels of neutralizing antibodies induced. Vaccinated animals protected against parenteral challenges with cell-free virus, but succumb and die on challenge with cell-associated virus (infected lymph nodes)
Inactivated AlHV-1 strain C500	Rabbit	1980 [45]	Inactivated cell-free virulent AlHV-1 C500
AlHV-1-like virus (707K virus) preparation isolated from clinical cases in American cattle	Cattle	1991 [47]	No protective immunity achieved with single or multiple rounds of vaccine administration. Cattle exposed to repeated inoculations developed MCF-like symptoms
Attenuated C500 strain-AlHV-1 from serially passaged cell cultures with Freund’s (unlicensed) adjuvant	Cattle	2008 [17]	Oro*-*nasal mucosal immunity induced in vaccinated cattle with high titres virus-neutralizing antibodies
Attenuated C500 strain-AlHV-1 from serially passaged cell cultures with Emulsigen (licensed) adjuvant	Cattle	2012 [48]	High titres of virus-neutralizing antibodies in both plasma and nasal secretions of vaccinated cattle. 6-month duration of protective immunity in vaccinated animals
Recombinant AlHV-1 virus with ORF73 deletion	Rabbit	2013 [50]	ORF73-deleted recombinant virus induced a strong antibody response. Animals protected against MCF-associated pathology following lethal challenge and with virulent virus
Attenuated C500 strain-AlHV-1 from serially passaged cell cultures with Emulsigen (licensed) adjuvant and unmethylated CpG oligodeoxy nucleotide (TLR9 agonist)	Cattle	2014 [49]	Unmethylated CpG oligodeoxynucleotide offers no additional advantage to length or level of protective immunity achieved using attenuated AlHV-1 with Emulsigen (licensed) adjuvant

The potential of virus preparations attenuated for virulence through serial passaging or deletion of virulent genes has been the focus of WA-MCF vaccine studies in the recent past. The performance of a live attenuated vaccine based on a high-pass attenuated C500 strain of AlHV-1 was assessed in cattle [17]. The attenuated vaccine, administered with Freund’s adjuvant intramuscularly, provided protective immunity against intranasal challenge with virulent AlHV-1. In a follow-up investigation, the attenuated vaccine gave a 6-month protection window when combined with Emulsigen, a licensed adjuvant, and administered to cattle by intramuscular inoculation [48, 49]. Another study by Palmeira and co-workers [50] demonstrated the application of recombinant AlHV-1 ORF73 null mutants, which lacked expression of the latency-associated nuclear antigen (LANA) homolog encoded by ORF73, as a promising recombinant vaccine candidate. Although not tested or validated in cattle, the ORF73 null mutant was apathogenic in rabbit models of WA-MCF and triggered a significant humoral response and protection from MCF-related pathology in vaccinated rabbits, conferring protection against subsequent infection with a virulent strain of AlHV-1.

The potential of these candidates as vaccines for WA-MCF appears promising but requires validation in cattle and other susceptible hosts. Presently, there is a paucity of data from large-scale field vaccine trials in locally-adapted cattle in eastern and southern Africa, which would be useful in validating promising candidates. Additionally, there remains a need for current vaccine studies to incorporate or identify markers to differentiate between infected and vaccinated animals (DIVA vaccine). DIVA vaccines incorporate or omit one or more detectable antigens, which then form the basis of distinguishing between natural challenge and vaccination by use of a companion serological or molecular diagnostic test. DIVA vaccines have been developed for a range of veterinary diseases affecting cattle and other livestock, as reviewed previously [51, 52]. Embracing a DIVA strategy for WA-MCF vaccine development would be useful in assessing the effectiveness of vaccination programmes, monitoring vaccine coverage and verifying breakthrough epidemics. The use of companion diagnostic tests alongside a DIVA vaccine would ensure compatibility between surveillance and vaccination programmes in working towards a comprehensive integrated control strategy for WA-MCF.

### The role of virus and host diversity in WA-MCF

The genome of AlHV-1, the causative agent of WA-MCF, was sequenced in 1997 [53]. Despite the availability of genome information for AlHV-1 and those of closely related MCF viruses [54, 55], genetic variation and evolution of AlHV-1 in wild and domestic hosts is poorly understood, as minimal attempts have been made to investigate viral diversity in clinical cases or in field isolates. However, a previous study by Shih and co-workers [56] outlined the existence of genetic variation in MCF viruses from different hosts based on restriction enzyme digestion patterns. In a recent study, the viral lytic transactivator protein gene (ORF50), the main diagnostic target in the nested PCR assay for AlHV-1, was well conserved among field samples in Tanzania, whereas a novel viral glycoprotein (A9.5) was found to be highly polymorphic at both the nucleotide and the protein level [5]. These observations indicate the need to investigate virus diversity in WA-MCF cases, because, while it is generally accepted that DNA viruses such as AlHV-1 may exhibit lower rates of mutation and genetic variation compared to RNA viruses, DNA viruses can accumulate point mutations and length polymorphisms, which may influence genetic variability, genome regulation and functions of key viral genes such as those responsible for virulence [57].

At present, it remains unknown if AlHV-1 is segregated into single or multiple genetic subtypes and whether there are any observable geographical patterns of distribution in eastern and southern Africa. The availability of powerful sequencing technologies presents fresh opportunities for genetic characterization of this important cattle pathogen. These technologies may be employed for spatial and temporal characterization of virus isolates from different outbreaks, geographical areas and hosts to provide insights into the genetic variability and evolution of the virus. This knowledge is crucial in vaccine trials and in guiding the design of diagnostic tests to diagnose all or at least the majority of the possible genetic variants.

The role of host genetics and immunity in WA-MCF has not been studied. A rich genetic diversity of locally adapted indigenous cattle exists in eastern and southern Africa where WA-MCF is present [58]. However, whether the genetic background of the cattle affects susceptibility or tolerance to AlHV-1 or plays a role in disease progression and pathology remains an area for future research. Previous studies of infections with related gammaherpesviruses have demonstrated a role for the host genotype in influencing immune responses during the course of infection [59]. There exists a need to understand both viral and host diversity and their interactions in immunological containment and pathology of WA-MCF. It will therefore be important to incorporate this knowledge in the design of diagnostic assays and in a rational WA-MCF vaccine development strategy.

## Conclusions

The control of WA-MCF in sub-Saharan Africa will be dependent on adoption of a comprehensive logical framework for integrated control as proposed in this review, rather than the present strategy of avoidance. While progress has been made over the decades towards understanding the etiology and pathology of this disease and development of a vaccine, there are persistent knowledge gaps concerning the role of virus and host diversity in clinical disease as well as opportunities for research on field-friendly diagnostic technologies. There is a need to harmonize resources towards research and a rational multi-dimensional strategy for integrated control, applicable to the regional context, without which WA-MCF will continue to be a threat not only to cattle production but also to wildlife conservation in eastern and southern Africa.
